# Percutaneous cyanoacrylate injection with endoscopic closure using an over-the-scope clip for refractory duodenocutaneous fistula

**DOI:** 10.1055/a-2590-2397

**Published:** 2025-05-19

**Authors:** Kento Shionoya, Shuntaro Mukai, Atsushi Sofuni, Takayoshi Tsuchiya, Reina Tanaka, Ryosuke Tonozuka, Takao Itoi

**Affiliations:** 1 Department Gastroenterology and Hepatology, Tokyo Medical University, Tokyo 160-0023, Japan

Over-the-scope (OTS) clip is useful for hemostasis in gastrointestinal bleeding or closure of gastrointestinal perforations or leaks 1 2 .

A 67-year-old woman with diabetes mellitus was presented to our hospital with abdominal pain. Abdominal computed tomography (CT) showed free air on the liver surface ( Fig. 1 ). Although the site of perforation could not be identified, emergency surgery was performed based on the diagnosis of upper gastrointestinal perforation. A perforation in the anterior wall of the duodenal bulb was found and closed with omental packing. On postoperative day 4, the drainage fluid became intestinal fluid-like, suggesting anastomotic leakage. Reoperation revealed separation of the anastomosis, making closure difficult. A jejunostomy was created, and a drain was placed percutaneously ( Fig. 2 ). Subsequent attempts to close the perforation failed due to persistent leakage, leading to an enlarged perforation and formation of a duodenocutaneous fistula. After nutritional management and improvement of the patient’s general condition, the perforation was closed, but the duodenocutaneous fistula persisted ( Fig. 3 and Fig. 4 ). Percutaneous injections of tissue adhesive were unsuccessful. A combined approach using percutaneous cyanoacrylate injection and endoscopic closure was performed.

**Fig. 1:**
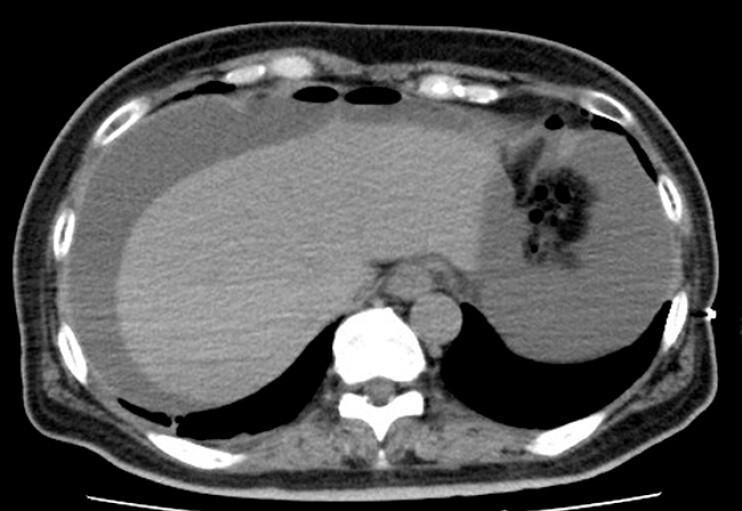
Abdominal CT image when visiting our institution. Abdominal CT showed free air and ascites on the liver surface. Abbreviation: CT, computed tomography.

**Fig. 2:**
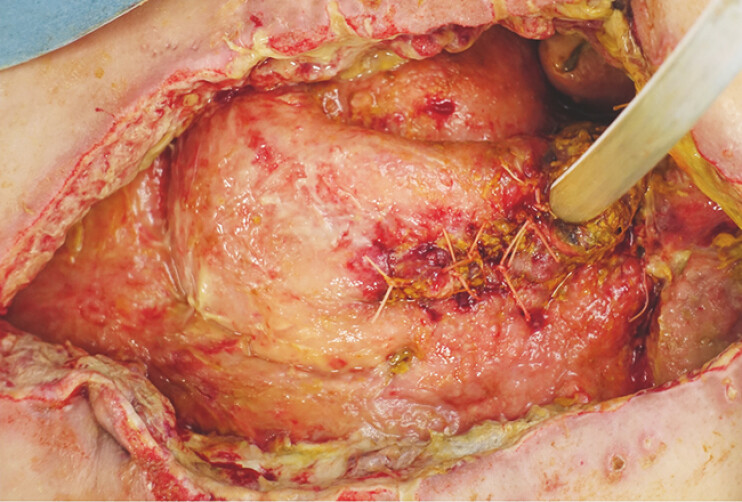
Intraoperative image at the time of reoperation. A jejunostomy was created, and a drain was placed percutaneously.

**Fig. 3:**
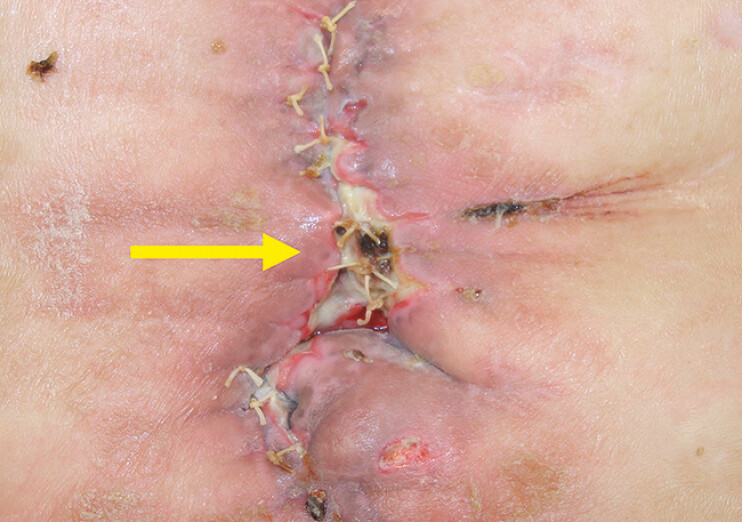
After reoperation image of body surface of the duodenocutaneous fistula. The duodenocutaneous fistula persisted after reoperation. (→) showed the persisted duodenocutaneous fistula.

**Fig. 4:**
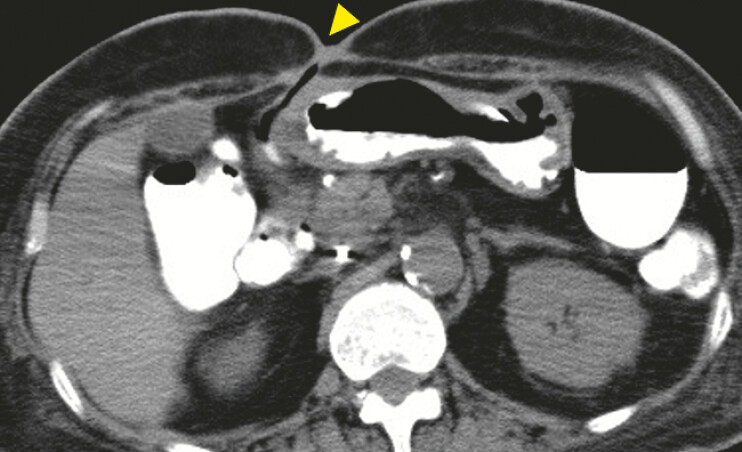
Abdominal CT image after reoperation. Abdominal CT showed the duodenocutaneous fistula persisted after reoperation. (△) showed the persisted duodenocutaneous fistula. Abbreviation: CT, computed tomography.

As cyanoacrylate administration is an off-label use, permission was obtained from the hospital ethics committee. The endoscope with the OTS clip attached was inserted, and the fistula was aspirated with the surrounding mucosa, though the mucosal surface was hard to aspirate. The clip was released after confirming sufficient aspiration, followed by percutaneous cyanoacrylate injection through the duodenal fistula site to complete the procedure ( Video 1 ). Two months postoperatively, CT of the abdomen showed no duodenocutaneous fistula, and the fistula was closed endoscopically ( Fig. 5 ).

Percutaneous cyanoacrylate injection combined with endoscopic closure using over the scope clip for refractory duodenocutaneous fistula.Video 1

**Fig. 5:**
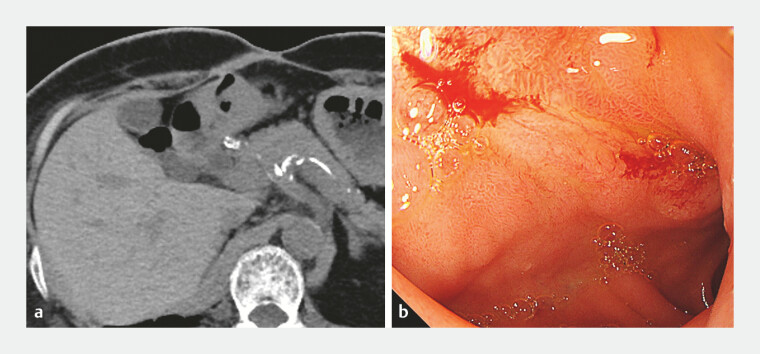
Abdominal image examination two months after endoscopic closure. **a** Abdominal CT image 2 months after endoscopic closure. Abdominal CT showed no duodenocutaneous fistula. **b** Endoscopic image 2 months after endoscopic closure. Endoscopic image showed the fistula was closed and the clip had dropped off. Abbreviation: CT, computed tomography.

Percutaneous cyanoacrylate injection combined with endoscopic duodenocutaneous fistula closure was effective for refractory duodenocutaneous fistula.

Endoscopy_UCTN_Code_TTT_1AO_2AI

## Notes

Endoscopy E-Videos https://eref.thieme.de/e-videos
*E-Videos* is an open access online section of the journal *Endoscopy* , reporting on interesting cases and new techniques in gastroenterological endoscopy. All papers include a high-quality video and are published with a Creative Commons CC-BY license. Endoscopy E-Videos qualify for HINARI discounts and waivers and eligibility is automatically checked during the submission process. We grant 100% waivers to articles whose corresponding authors are based in Group A countries and 50% waivers to those who are based in Group B countries as classified by Research4Life (see: https://www.research4life.org/access/eligibility/ ). This section has its own submission website at https://mc.manuscriptcentral.com/e-videos .

## References

[1] Mangas-Sanjuan C Martínez-Moreno B Bozhychko M Over-the-scope clip for acute esophageal variceal bleeding Dig Endosc 2019 31 712 716 10.1111/den.13493 31330068

[2] Tashima T Nonaka K Ryozawa S Unprecedented problems and troubleshooting during over the-scope clip system use: Suction error and suture cutting procedure Dig Endosc 2019 31 e36 e37 10.1111/den.13309 30500106

